# Metabolomics Analysis Reveals Drought Responses of Trifoliate Orange by Arbuscular Mycorrhizal Fungi With a Focus on Terpenoid Profile

**DOI:** 10.3389/fpls.2021.740524

**Published:** 2021-10-06

**Authors:** Sheng-Min Liang, Fei Zhang, Ying-Ning Zou, Kamil Kuča, Qiang-Sheng Wu

**Affiliations:** ^1^College of Horticulture and Gardening, Yangtze University, Jingzhou, China; ^2^College of Biology and Agricultural Resources, Huanggang Normal University, Huanggang, China; ^3^Department of Chemistry, Faculty of Science, University of Hradec Kralove, Hradec Kralove, Czechia

**Keywords:** citrus, metabolite, mycorrhiza, terpenoid, water stress

## Abstract

Soil water deficit seriously affects crop production, and soil arbuscular mycorrhizal fungi (AMF) enhance drought tolerance in crops by unclear mechanisms. Our study aimed to analyze changes in non-targeted metabolomics in roots of trifoliate orange (*Poncirus trifoliata*) seedlings under well-watered and soil drought after inoculation with *Rhizophagus intraradices*, with a focus on terpenoid profile. Root mycorrhizal fungal colonization varied from 70% under soil drought to 85% under soil well-watered, and shoot and root biomass was increased by AMF inoculation, independent of soil water regimes. A total of 643 secondary metabolites in roots were examined, and 210 and 105 differential metabolites were regulated by mycorrhizal fungi under normal water and drought stress, along with 88 and 17 metabolites being up-and down-regulated under drought conditions, respectively. KEGG annotation analysis of differential metabolites showed 38 and 36 metabolic pathways by mycorrhizal inoculation under normal water and drought stress conditions, respectively. Among them, 33 metabolic pathways for mycorrhization under drought stress included purine metabolism, pyrimidine metabolism, alanine, aspartate and glutamate metabolism, etc. We also identified 10 terpenoid substances, namely albiflorin, artemisinin (−)-camphor, capsanthin, β-caryophyllene, limonin, phytol, roseoside, sweroside, and α-terpineol. AMF colonization triggered the decline of almost all differential terpenoids, except for β-caryophyllene, which was up-regulated by mycorrhizas under drought, suggesting potential increase in volatile organic compounds to initiate plant defense responses. This study provided an overview of AMF-induced metabolites and metabolic pathways in plants under drought, focusing on the terpenoid profile.

## Introduction

Crops are often subjected to abiotic stresses in the process of the growth and development, while abiotic stress also activates relevant functional genes and metabolic pathways to mitigate the stress damage of crops (Giordano et al., 2021). Plant stress regulation is a complex network, and it is difficult to fully analyze the responsive mechanisms of plants to stress by studying a single gene or metabolic pathway alone (Li et al., 2017; Yang et al., 2021). Metabolomics is a powerful tool to assess the whole change of organisms at the metabolite level (Alseekh et al., 2018) and also is essential for understanding the chemical signals during plant growth and development (Carrera et al., 2021). In the analysis of metabolomics, metabolites are small molecules that are formed and/or altered during metabolisms, and changes of metabolite profiles can establish a close relationship between genotype and phenotype and also reveal the plant–environment interaction comprehensively and systematically (Bernardo et al., 2019; Carrera et al., 2021).

Arbuscular mycorrhizal fungi (AMF), subphylum Glomeromycotina of the phylum Mucoromycota, are the most widely distributed mycorrhizal fungi in soil, and can colonize the root of host plants to establish arbuscular mycorrhizal symbionts (Kokkoris et al., 2020). Mycorrhizal fungi establish a well-developed network of extraradical mycelium outside the root system, which is directly involved in water and nutrient uptake and therefore responds to various environmental stresses including drought (Meng et al., 2020; Malhi et al., 2021). AMF-induced enhancement of drought tolerance is involved in many metabolic processes, such as proline, indoleacetic acid, polyamines, fatty acids, aquaporins, antioxidant defense system, H^+^-ATPase, rhizospheric microenvironment, and so on (Wu et al., 2019; He et al., 2020; Khalil and El-Ansary, 2020; Sheteiwy et al., 2021; Zou et al., 2021; Cheng et al., 2021a,b,c). Metabolomics analysis has been applied to reveal mycorrhiza-associated metabolite changes following AMF inoculation (Rivero et al., 2015). However, very few studies have attempted to use metabolomics tool to analyze metabolite changes caused by AMF inoculation under abiotic stress (e.g., drought and salinity) conditions (Rivero et al., 2018; Bernardo et al., 2019; Yang et al., 2020). For example, Yang et al. (2020) revealed that under saline conditions, inoculation with *Rhizophagus intraradices* induced changes in amino acids, organic acids, flavonoids, sterols, and hormones in *Puccinellia tenuiflora* plants to improve osmoregulation, maintain cell membrane stability, and enhance antioxidant systems. In wheat, *Funneliformis mosseae* mainly triggered sugars, lipids, and oleuropein lactones involved in stress mechanisms under drought stress (Bernardo et al., 2019). These limited studies still revealed that arbuscular mycorrhizal symbionts responded to stress by regulating metabolic plasticity (Rivero et al., 2018).

Terpenoids are the most diverse and widely distributed class of secondary metabolites present in plants. Terpenoids are volatile, serve as mediators of communication between plants and other organisms, and are biologically and ecologically important for the plants’ own reproduction and defense (Jiang, 2012). They guarantee the survival of plants in stressed environments by performing several functions, such as protecting plant tissues from pathogens or herbivores and helping reproduction by attracting pollinators or seed dispersers (Quan, 2013). Severe drought dramatically increased total monoterpenes and resin acids in *Pinus sylvestris* and *Picea abies* seedlings (Turtola et al., 2003). In an aromatic plant *Tanacetum vulgare*, root terpenoids were obviously induced by drought, and such increase in root terpenoids would serve as more storage of resources for regrowth (Kleine and Müller, 2014).

Earlier studies have shown that secondary metabolites of many hosts such as terpenoids, flavonoids, and alkaloids were regulated by AMF inoculation under drought conditions (Bernardo et al., 2019). In *P*. *tenuiflora* seedlings, AMF inoculation under saline conditions significantly altered the relative concentrations of various metabolites, including amino acids, amines, carbohydrates, polyols, phytohormone, steroids, nucleic acids, fatty acids, flavonoids, organic acids, but terpenoids were not reported (Yang et al., 2020). However, in two wheat cultivars, *F*. *mossea*e caused the change of terpenoids, along with the up-regulation of a limonene-1,2-doil and (20S)-ginsenoside Rh2 and the down-regulation of both 7-deoxyloganate and hemigossypol-6-methyl ether (Bernardo et al., 2019). Kaur and Suseela (2020) reviewed the changes in primary and secondary metabolites of plants induced by mycorrhizal fungi and concluded that host terpene metabolism is specific to the host-associated AMF and terpene species. It was shown that sesquiterpenoid cyclohexenone derivatives could be induced to increase by *Glomus intraradices* (Maier et al., 1997) in the member of Poaceae, but sesquiterpenoid cyclohexenone glycoside was not induced by AMF under biotic and abiotic stresses. Various volatile terpenoids in host plants were also induced by AMF, resulting in an increased emission of these compounds in response to the environment (Rapparini et al., 2007; Fontana et al., 2009). It seems that terpenoids could be regulated by AMF inoculation under stress. Because of the wide variety of terpenoids, metabolomics is the way to clearly outline the response pattern for mycorrhizal inoculation under drought conditions, while how mycorrhizas respond to drought through changes of terpenoid profile is unclear.

The aim of this study was to comprehensively analyze the changes in the metabolome of citrus (a drought-sensitive industrial crop) by AMF inoculation under soil well-watered and soil drought stress through non-targeted metabolomics, with a focus on the response of terpenoids in the differential metabolites to reveal the mechanisms by which mycorrhizas enhance plant drought tolerance.

## Materials and Methods

### Mycorrhizal Inoculum

An arbuscular mycorrhizal fungus, *R*. *intraradices* (N.C. Schenck & G.S. Smith) C. Walker & A. Schüßler, was used. The mycorrhizal fungal strain (FLR12, Myke^®^PRO) was provided by the Premier Tech Ltd. (Quebec, Canada), which is a company for the production of commercial mycorrhizal inoculants. The Myke FLR12 strain of *R*. *intraradices* was designed for the improved growth of annuals and perennials and also presented positive effects on plant growth performance of trifoliate orange (*Poncirus trifoliata* L. Raf., a citrus rootstock; Cheng et al., 2019). The purchased fungal strain was proliferated by employing white clover as the host plant in pots under the condition of 900μmol/m^2^/s photo density, 28°C/20°C day/night temperatures, and 68% relative air humidity. After 3months, the white clover plants were harvested, the aboveground parts were removed, and the roots and potting substrates were collected. The roots were cut, mixed well with the growth substrate, dried naturally for 5days, and collected as mycorrhizal fungal inoculum. Thus, the mycorrhizal inoculum contained AMF-colonized root segments, spores, sporocarps, and mycorrhizal mycelium. Harvested inocula were kept at 4°C for a maximum of 4months. Prior to the application of AMF inocula, we used sucrose gradient centrifugation (Brundrett et al., 1994) to isolate spores in the inocula and observed 19 spores/g inoculum.

### Experimental Design

A 2^2^ experimental design was used for this experiment. One factor was AMF inoculations with and without *R*. *intraradices*; the other factor was soil water regimes, including well-watered (75% of maximum field water holding capacity) and drought stress (55% of maximum field water holding capacity). As a result, there were four treatments in the experiment: the non-inoculated seedlings (non-AMF) under well-watered, the inoculated seedlings (AMF) under well-watered, the non-inoculated seedlings under drought stress, and the inoculated seedlings under drought stress. Each treatment was replicated 8 times, with a total of 32 pots.

### Plant Culture

Trifoliate orange seeds were sterilized by 75% alcohol solutions for 5min, rinsed with distilled water three times, and germinated in sterile sand in an incubator at 28°C/20°C day/night temperature and relative air humidity of 80%. When the seedlings developed four leaves, the seedlings were transplanted into a 2.3-L plastic pot pre-filled with 2.8kg of autoclaved mixture with soil and sand (1:1, v:v). At transplanting, 100g of mycorrhizal fungal inoculums was applied into the pot as the AMF treatment, and the non-AMF treatment was correspondingly mixed with an equal amount of autoclaved mycorrhizal inocula. After transplanting, these seedlings were exposed to the condition of 900μmol/m^2^/s photo density, 28°C/20°C day/night temperatures, and 68% relative air humidity and also grew in soil well-watered regime for 11weeks before soil drought treatment. 3days prior to soil drought stress, selected pots were stopped from watering and allowed to reach the designed soil moisture content for drought stress. Subsequently, half of the inoculated and non-inoculated plants were subjected to soil drought stress treatment for 9weeks, and the remaining plants were still grown under soil well-watered regime conditions for 9 weeks. The reduced water of pots was supplemented by weighing daily at 18:00pm. This study was conducted for 20weeks.

### Determination of Root Mycorrhizal Colonization and Plant Biomass

At harvest plants were divided into shoots and roots, weighted and frozen with liquid nitrogen and immediately stored at−80°C for subsequent analysis.

Five 1-cm-long root segments were cut from each plant, cleared in 10% KOH solution at 95°C for 1.5h, and stained with 0.05% trypan blue in lactophenol (Phillips and Hayman, 1970). The arbuscular mycorrhizal structure of roots was observed under a light microscope, and the root mycorrhizal colonization rate was estimated according to the following formula:

Extraction of Metabolome Samples

Root tissues were vacuum freeze-dried, and the dried samples were ground into a homogenous powder. Subsequently, 100mg of the powder was extracted with 70% methanol at 4°C overnight, vortexing and shaking three times during the extracted process. The samples were centrifuged at 10,000×g/min for 10min, and the supernatant was filtered through a microporous membrane (0.22μm), followed by LC–MS/MS analysis.

### LC–MS/MS Analysis

Data acquisition systems included Ultra Performance Liquid Chromatography (UPLC; Shim-pack UFLC SHIMADZU CBM20A, Shimadzu Corporation, Kyoto, Japan^1^) and tandem mass spectrometry (Applied Biosystems 6,500 QTRAP, Thermo Fisher Scientific, Waltham, United States).^2^ Analytical conditions were as follows: (1) Chromatographic column was Waters ACQUITY UPLC HSS T3 C18 with 1.8μm and 2.1mm×100mm; (2) mobile phase consisted of ultrapure water (with 0.04% acetic acid added) for the aqueous phase and acetonitrile (with 0.04% acetic acid added) for the organic phase; (3) elution gradient consisted of water and acetonitrile with 95:5 (v/v) at 0–10min, 5:95 at 11–12min, 95:5 at 13–15min; and (4) a flow rate of 0.4ml/min, a column temperature of 40°C, and an injection volume of 5μl.

In the LC–MS/MS system, the main parameters of the linear ion trap and triple quadrupole included: the electrospray ionization at 550°C, the mass spectrometry at 5500V, the curtain gas at 25psi, the collision-activated dissociation parameters at a high grade. In the triple quadrupole, each ion pair was scanned and detected according to the optimized declustering potential and collision energy. The assay data were analyzed and processed using the Analyst 1.6.3 (AB Sciex, Foster, California, United States) software.

### Metabolite Characterization and Quantification

Based on the self-built metware database and the public database of metabolite information, the primary and secondary spectra of mass spectrometry were analyzed qualitatively. The public database of metabolites included the MassBank,^3^ the KNAPSAcK,^4^ the HMDB,^5^ the MoTo DB,^6^ and the METLIN.^7^ Metabolite quantification was accomplished using the multiple reaction monitoring mode of triple quadrupole mass spectrometry. After obtaining the metabolite spectra for each sample, the peak areas of the mass spectra of all substances were integrated and corrected for the mass spectra of the same metabolite in different samples. The data were formatted in logarithmic transformation plus centralization using a SIMCA software (V14.1, Sartorius Stedim Data Analytics AB, Umea, Sweden) and then analyzed by automated modeling (Wiklund et al., 2008).

### Analysis of Metabolites

Principal component analysis (PCA) was carried out by SIMCA-P software (Umetrics AB, Kinnelon, NJ, United States). The statistical method of orthogonal projections to latent structures-discriminant analysis (OPLS-DA) was utilized to analyze the results of metabolites and to obtain more credible information about the correlation between metabolite group differences and experimental comparison groups. The OPLS-DA modeling analysis was performed on the first principal component. The validity of the model was assessed by the RY (interpretability of the model for categorical variable Y) and Q (predictability of the model) obtained after cross-validation.

### Differential Metabolite Screening

By considering the results of both multivariate and univariate statistical analysis methods, we observed the data from different perspectives and draw conclusions, in order to avoid false positive errors or model overfitting caused by using only one type of statistical analysis method. The differential metabolites were defined as per the Student’s t-test at *p*<0.05, along with >1 of the variable importance in the projection of the first principal component of the OPLS-DA model.

### KEGG Annotation of Differential Metabolites and Metabolic Pathway Analysis

In this study, all the pathways mapped by the differential metabolites of *Citrus sinensis*, including energy metabolism, substance transport, signaling, and cell cycle regulation, were compiled, in terms of the Kyoto Encyclopedia of Genes and Genomes Pathway database.^8^

### Statistical Analysis

Data were analyzed for ANOVA using the SAS software. Bonferroni correction test at 0.05 levels was used to compare the significant differences between treatments.

## Results

### Changes in Mycorrhizal Growth

**Figure 1:**
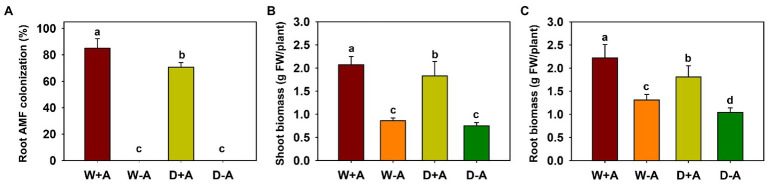
Changes in root mycorrhizal colonization **(A)**, shoot biomass **(B)**, and root biomass **(C)** of trifoliate orange seedlings under well-watered and drought stress conditions after inoculation with *Rhizophagus intraradices*. Different letters above the bar indicate significant differences at *p*<0.05. Abbreviation: W + A, inoculation with *R*. *intraradices* under well-watered conditions; W-A, inoculation without *R*. *intraradices* under well-watered conditions; D + A, inoculation with *R*. *intraradices* under drought stress conditions; D-A, inoculation without *R*. *intraradices* under drought stress conditions.

No mycorrhizal colonization was observed in any of the roots of the non-inoculated trifoliate orange plants, and the root colonization of inoculated plants varied from 70 to 85% (Figure 1A). The soil drought treatment significantly reduced root AMF colonization by 17.6%, compared to the well-watered treatment.

### Changes in Plant Biomass

Drought inhibited root biomass production of inoculated trifoliate orange seedlings, whereas inoculation with AMF significantly promoted shoot and root biomass, independent of soil moisture conditions (Figures 1B,C). Compared to non-AMF inoculation, *R*. *intraradices* inoculation significantly increased shoot and root biomass, as evidenced by a significant increase of 141.27 and 68.52% in shoot and root biomass, respectively, under well-watered conditions and by an increase of 145.89 and 74.11% under drought conditions, respectively.

### PCA Analysis of Root Metabolites

**Figure 2:**
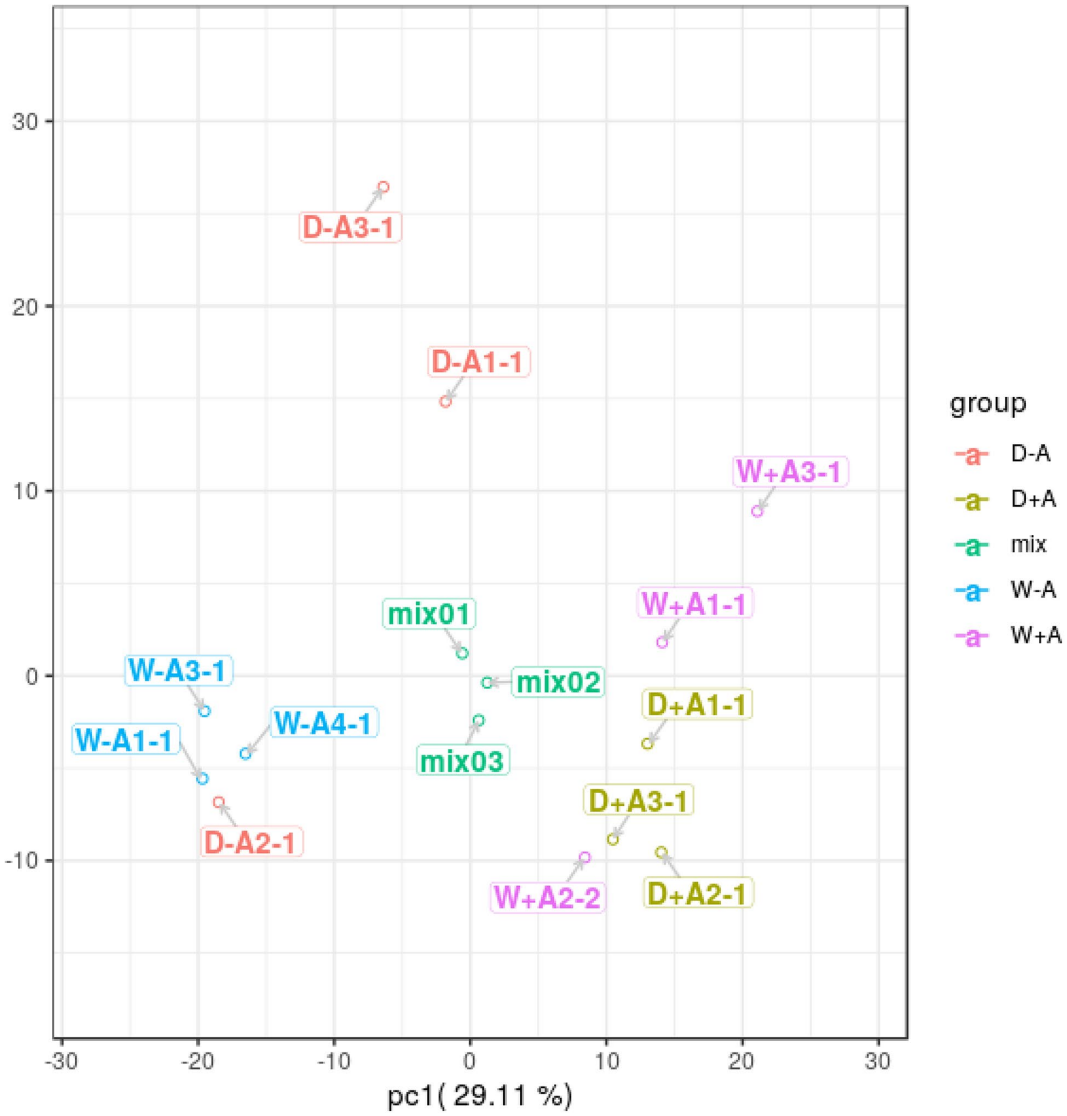
Principal component analysis of metabolites in roots of trifoliate orange seedlings under well-watered and drought stress conditions after inoculation with *Rhizophagus intraradices*. Abbreviation: W + A, inoculation with *R*. *intraradices* under well-watered conditions; W-A, inoculation without *R*. *intraradices* under well-watered conditions; D + A, inoculation with *R*. *intraradices* under drought stress conditions; D-A, inoculation without *R*. *intraradices* under drought stress conditions; mix, quality control samples.

A total of 643 secondary metabolites in roots were found in this study. PCA was performed on the quantitative results of metabolites from these samples (including quality control samples; Figure 2). A total of 2 principal components were obtained, where the X-axis represented the first principal component, and the Y-axis represented the second principal component. The quality control samples (mix) were clustered together, indicating that the sample mass spectrometry monitoring analysis was stable and the data were reproducible and credible. Samples were clustered across treatments, with the exception of a sample from non-inoculated seedlings under drought stress and a sample from inoculated seedlings under well-watered, which were deviant, probably due to differences in the samples themselves. However, in general, the sample treatments were clearly differentiated, with the first principal component being able to clearly distinguish between AMF inoculation and non-AMF inoculation.

### Orthogonal Partial Least Squares-Discriminant Analysis

**Figure 3:**
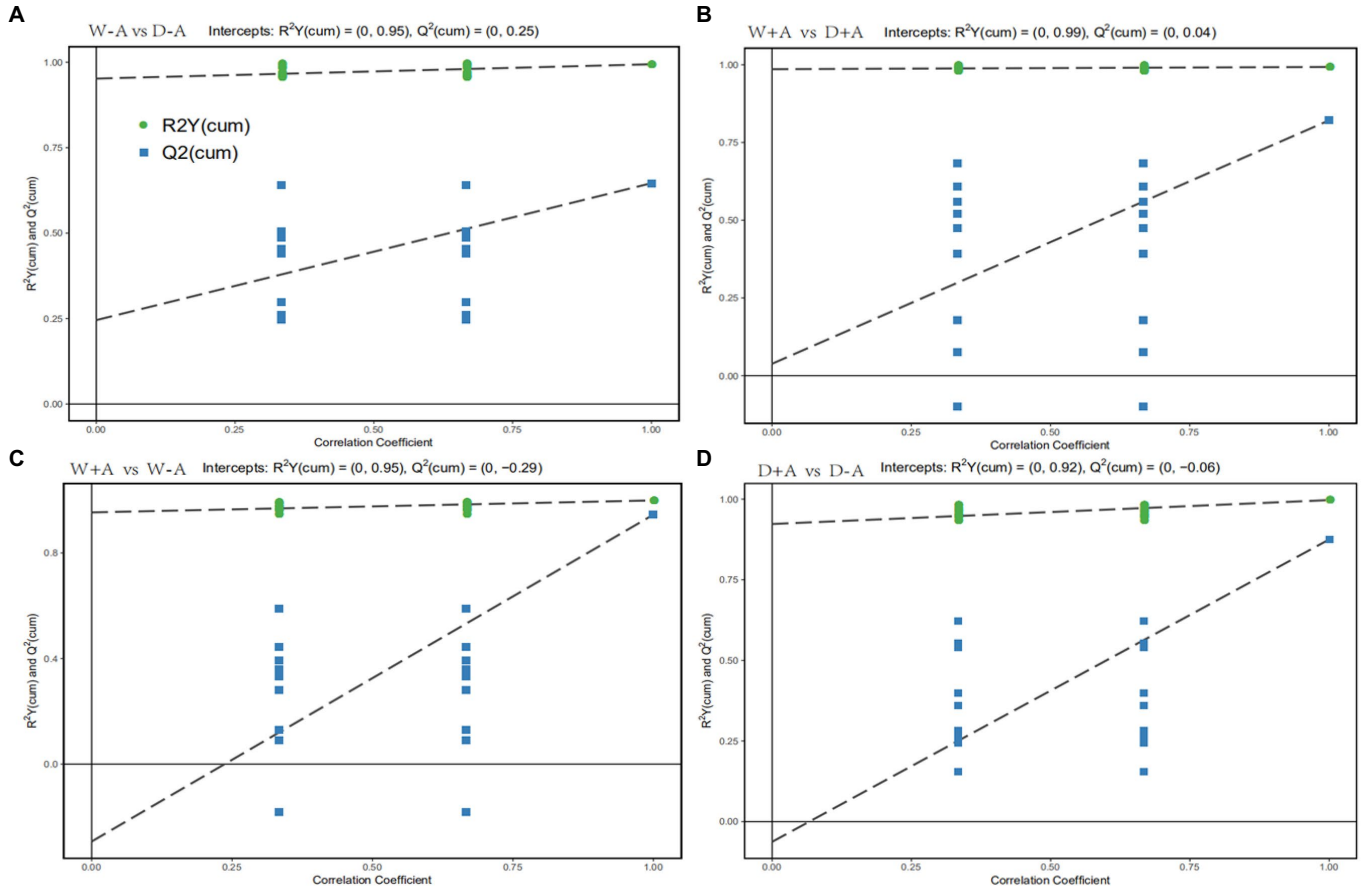
OPLS-DA of metabolites in roots of trifoliate orange seedlings under well-watered and drought stress conditions after inoculation with *Rhizophagus intraradices*. Abbreviation: W + A, inoculation with *R*. *intraradices* under well-watered conditions; W-A, inoculation without *R*. *intraradices* under well-watered conditions; D + A, inoculation with *R*. *intraradices* under drought stress conditions; D-A, inoculation without *R*. *intraradices* under drought stress conditions; mix, quality control samples.

Orthogonal Partial Least Squares-Discriminant Analysis is a regression method based on characteristic variables. Based on the analysis of OPLS-DA, we obtained reliable information on the degree of correlation between group differences in metabolites and experimental groups, except the orthogonal variables in metabolites not correlated with categorical variables (Figure 3). The slope of the OPLS-DA model was positive, and R2Y was close to 1 and Q2 was close to 0 for both the comparison between well-watered and drought stress treatments and between inoculations with AMF and non-AMF, indicating that the model was stable. This indicated that for the results of this metabolomic test, the treatments were grouped very well with significant differences between groups.

### Screening for Differential Metabolites in Roots

**Figure 4:**
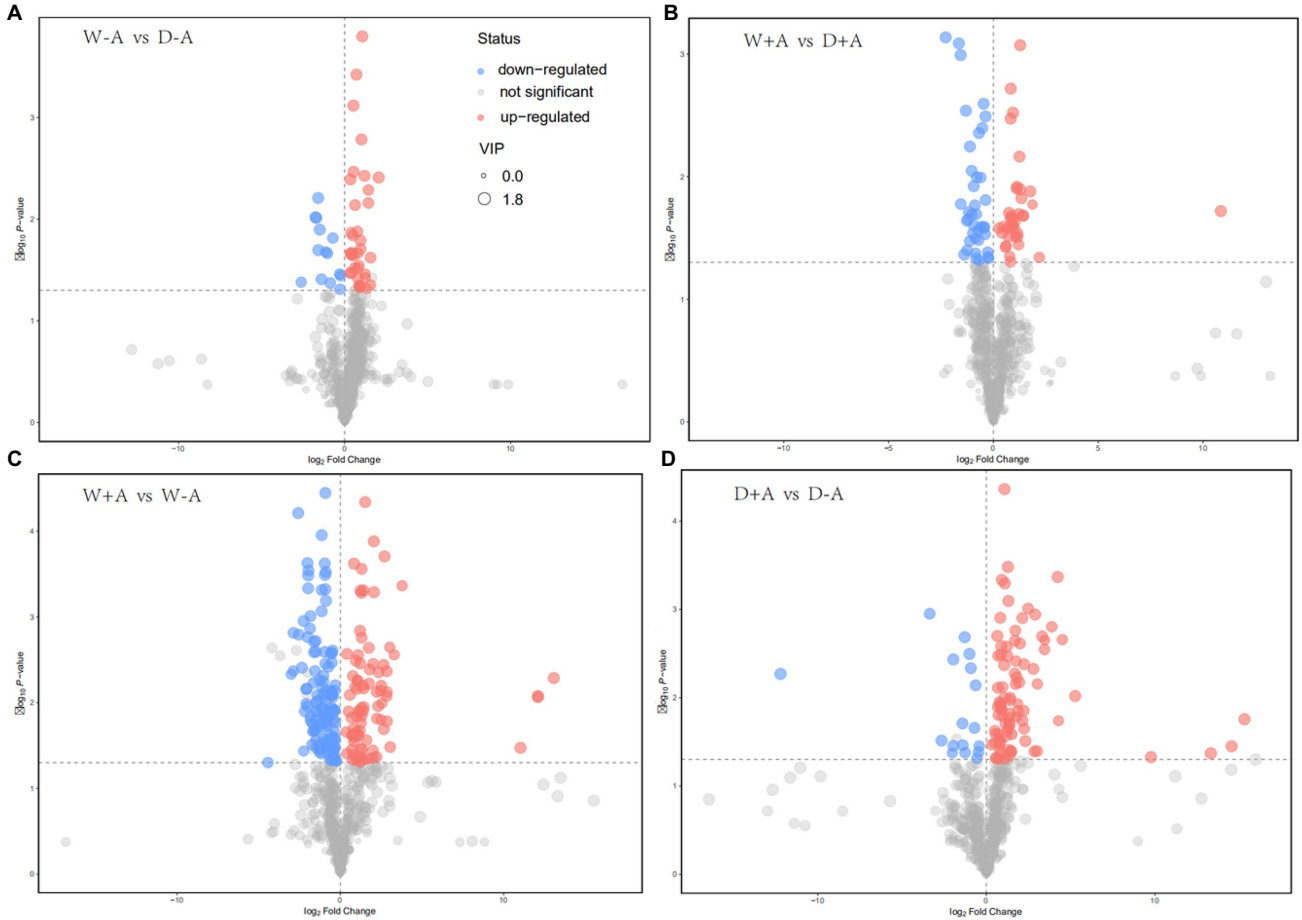
Volcanic map of different metabolites in roots of trifoliate orange seedlings under well-watered and drought stress conditions after inoculation with *Rhizophagus intraradices*. Abbreviation: W + A, inoculation with *R*. *intraradices* under well-watered conditions; W-A, inoculation without *R*. *intraradices* under well-watered conditions; D + A, inoculation with *R*. *intraradices* under drought stress conditions; D-A, inoculation without *R*. *intraradices* under drought stress conditions; mix, quality control samples.

A total of 643 metabolites were compared and screened for differential metabolites between treatments (screening conditions: VIP>1; *p*<0.05). The 50 differential metabolites were screened in the non-inoculated seedlings under drought stress versus well-watered, of which 14 differential metabolites were significantly up-regulated and 36 metabolites were significantly down-regulated (Figure 4A). Similarly, 76 differential metabolites were screened in the inoculated seedlings under drought stress versus well-watered, of which 39 metabolites were significantly up-regulated and 37 metabolites were down-regulated (Figure 4B). We also screened 210 differential metabolites under well-watered conditions after AMF inoculation, of which 86 metabolites were substantially up-regulated and 124 metabolites were down-regulated (Figure 4C). Under drought stress conditions, AMF inoculation caused 105 differential metabolites, of which 88 and 17 metabolites were up-regulated and down-regulated, respectively (Figure 4D).

### KEGG Annotation of Differential Metabolites in Roots

KEGG annotation analysis of differential metabolites showed four, thirty-six, thirty-eight, and thirty-six differential metabolic pathways in the non-inoculated seedlings under drought stress versus well-watered, the inoculated seedlings under drought stress versus well-watered, under well-watered conditions by AMF inoculation versus non-AMF inoculation, and under drought stress conditions by AMF inoculation versus non-AMF inoculation, respectively (Supplementary Figures 1–4). Under well-watered conditions, AMF inoculation caused the differential metabolites associated with ABC transporters, biosynthesis of amino acids, nicotinate and nicotinamide metabolism, glycine, serine and threonine metabolism, arginine and proline metabolism, amino sugar and nucleotide sugar metabolism, cysteine and methionine metabolism, histidine metabolism, tryptophan metabolism, beta-alanine metabolism, photosynthesis, arginine biosynthesis, alanine, aspartate and glutamate metabolism, valine, leucine and isoleucine biosynthesis, lysine degradation, tyrosine metabolism, etc (Supplementary Figure 3). Under drought stress conditions, AMF triggered differential metabolites involved in biosynthesis of amino acids, arginine biosynthesis, glycine, serine and threonine metabolism, cysteine and methionine metabolism, arginine and proline metabolism, niacin and nicotinamide metabolism, lysine degradation, histidine metabolism, tyrosine metabolism, carbon fixation in photosynthetic organisms, N metabolism, etc (Supplementary Figure 4).

### Analysis of Metabolic Pathways in Differential Metabolites in Roots

**Figure 5:**
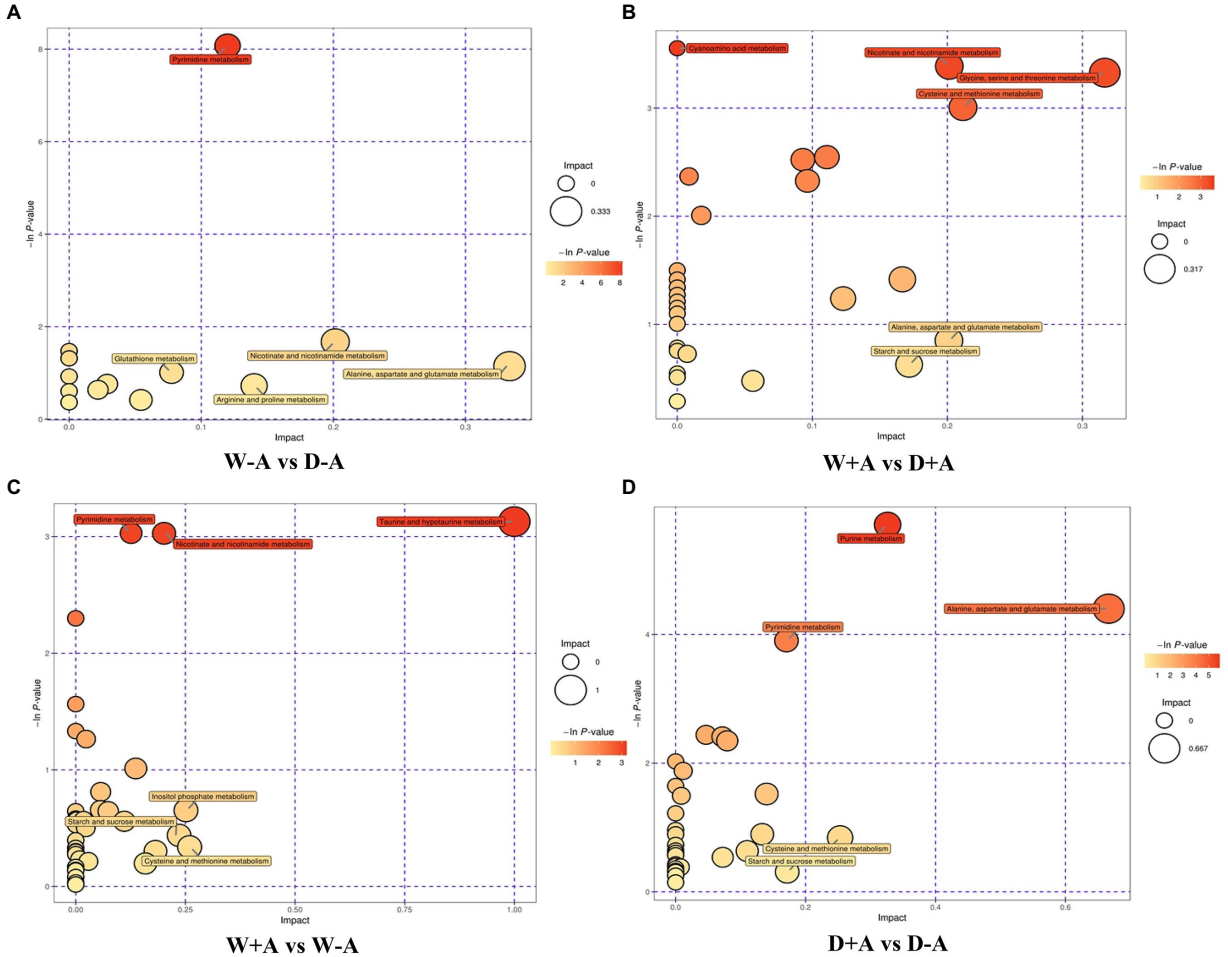
Bubble chart of pathway analysis of different metabolites in different treatment groups of trifoliate orange seedlings under well-watered and drought stress conditions after inoculation with *Rhizophagus intraradices*. Abbreviation: W + A, inoculation with *R*. *intraradices* under well-watered conditions; W-A, inoculation without *R*. *intraradices* under well-watered conditions; D + A, inoculation with *R*. *intraradices* under drought stress conditions; D-A, inoculation without *R*. *intraradices* under drought stress conditions; mix, quality control samples.

In this study, through comprehensive analysis (including enrichment analysis and topological analysis) of the pathways in which the differential metabolites were located, further screening of the pathways could be conducted to find the key pathways with the highest correlation with the difference of metabolites. Based on the comprehensive analysis, differential metabolites of non-inoculated plants under drought stress versus well-watered conditions were mainly annotated to fourteen metabolic pathways, including pyrimidine metabolism, nicotinate and nicotinamide metabolism, glutathione metabolism, arginine and proline metabolism, alanine, aspartate and glutamate metabolism, etc., among them L-glutamic acid was involved in seven metabolic pathways simultaneously (Figure 5A). In the inoculated seedlings, compared with well-watered treatment, drought treatment triggered differential metabolites annotated to thirty-one metabolic pathways (e.g., starch and sucrose metabolism and alanine, aspartate and glutamate metabolism), of which L-aspartic acid was involved in ten of these metabolic pathways as a differential metabolite (Figure 5B). Under well-watered conditions, mycorrhizal inoculation caused the differential metabolites annotated to thirty-eight metabolic pathways including pyrimidine metabolism, taurine and hypotaurine metabolism, inositol phosphate metabolism, etc (Figure 5C). Under drought stress conditions, the differential metabolites were annotated to thirty-three metabolic pathways for mycorrhization compared to non-mycorrhization, including purine metabolism, pyrimidine metabolism, alanine, aspartate and glutamate metabolism, etc (Figure 5D).

### Changes in Terpenoid Profiles in Roots

Among the differential metabolites, we identified ten terpenoid substances, namely albiflorin, artemisinin (−)-camphor, capsanthin, β-caryophyllene, limonin, phytol, roseoside, sweroside, and α-terpineol, with sweroside belonging to terene and the other nine to terpene (Table 1). We also found that in non-inoculated plants, drought triggered a significant accumulation of phytol up to 2.62-fold, but significantly inhibited the relative concentrations of capsanthin, β-caryophyllene, and α-terpineol up to 0.58–0.76-fold; in inoculated plants, soil drought only triggered a significant accumulation of sweroside up to 1.63-fold. In addition, under well-watered conditions, AMF inoculation significantly reduced relative concentrations of capsanthin, α-terpineol, sweroside, limonin, roseoside, albiflorin (−)-camphor, and artemisinin; however, under drought stress conditions, AMF inoculation significantly increased relative concentration of β-caryophyllene, up to 1.78-fold, but significantly reduced relative concentration of phytol, up to 0.38-fold.

**Table 1:** Changes in relative concentration and fold changes of terpenoids in differential metabolites in roots of trifoliate orange under well-watered (WW) and drought stress (DS) after AMF inoculation.

Comparison of treatments	Terpenoids	Category	KEEG ID	Ionization model	*p*-value	VIP	Fold change
DS vs. WW in non-AMF seedlings	Phytol	Terpene	C01389	[M-H]-	0.039	1.70	2.62↑
	Capsanthin	Terpene	C08584	[M+H]+	0.034	1.55	0.76↓
	β-Caryophyllene	Terpene	C09629	[M+H]+	0.038	1.61	0.58↓
	α-Terpineol	Terpene	C16772	[M+H]+	0.014	1.66	0.71↓
DS vs. WW in AMF seedlings	Sweroside	Terene	C17071	[M+H]+	0.027	1.57	1.63↑
AMF vs. non-AMF under WW	Limonin	Terpene	C03514	[M+H]+	0.003	1.39	0.52↓
	Roseoside	Terpene	–	[M+H]+	0.002	1.32	0.17↓
	Sweroside	Terpene	C17071	[M+H]+	0.008	1.39	0.38↓
	Albiflorin	Terpene	C17457	[M+H]+	0.014	1.30	0.50↓
	(−)-Camphor	Terpene	C00809	[M+H]+	0.025	1.21	0.79↓
	Capsanthin	Terpene	C08584	[M+H]+	0.027	1.22	0.80↓
	Artemisinin	Terpene	C09538	[M+H]+	0.032	1.21	0.61↓
	α-Terpineol	Terpene	C16772	[M+H]+	0.008	1.29	0.70↓
AMF vs. non-AMF under DS	Phytol	Terpene	C01389	[M-H]-	0.034	1.53	0.38↓
	β-Caryophyllene	Terpene	C09629	[M+H]+	0.001	1.60	1.78↑

*↑ and ↓ indicate that the variable is increased or decreased*.

## Discussion

An important function of arbuscular mycorrhizal symbiosis is to accelerate the growth of the host plant, both under normal and unfavorable environmental conditions (Malhi et al., 2021). Our study also confirmed that *R*. *intraradices* strongly promoted shoot and root biomass production in trifoliate orange regardless of soil moisture content, and the promoting effect was relatively higher under drought conditions than under well-watered conditions. Zhang et al. (2018) used a device to separate mycorrhizal extraradical hyphae from inoculated trifoliate orange rhizosphere for quantitative estimation of hyphal water absorption rate and found that the hyphal water absorption rate was much higher under soil moisture-limited conditions than under soil moisture-sufficient conditions. This suggests that the improved plant growth of AMF is more important on arid soil than on saturated soil and therefore more attention needs to be paid to mycorrhizas in arid zones (Allen, 2006).

Metabolomic analyses implied changes in root metabolites of trifoliate orange seedlings in response to AMF inoculation. Among them, in non-inoculated plants, 50 differential metabolites were identified and metabolites were predominantly down-regulated. Similarly, in quinoa (without AMF inoculation; *Chenopodium quinoa*), drought stress triggered 60 differential metabolites, 53 of which were down-regulated (Shi et al., 2020); in tall fescue (*Festuca arundinacea*), drought stress caused 282 differential metabolites, 148 of which were down-regulated (Li et al., 2017). However, in the inoculated trifoliate orange plants, 76 differential metabolites were identified, and these metabolites were predominantly up-regulated by drought stress. This result implied an elevated number of differential metabolites and a reversal of the metabolite response pattern from down-regulation (non-inoculated plants) to up-regulation (inoculated plants). In the analysis of differential metabolite pathway, drought-induced differential metabolites of non-inoculated plants and inoculated plants were annotated to 14 and 31 metabolic pathways, respectively. Among them, non-inoculated plants were dominated by amino acid (e.g., L-glutamic acid) metabolism and inoculated plants were dominated by sugars and amino acids (e.g., L-aspartic acid), showing the different metabolic responses of both. Thus, inoculated plants have stronger metabolic activity in response to drought stress than non-inoculated plants by means of predominant up-regulation, further indicating a higher drought tolerance potential of mycorrhizal plants versus non-mycorrhizal plants.

In addition, the presence of mycorrhizal symbionts strongly stimulated the changes of metabolites in roots of trifoliate orange under well-watered and drought stress, showing, respectively, 210 and 105 differential metabolites, which were predominantly down-regulated under well-watered and up-regulated under soil drought. Yang et al. (2020) also reported more up-regulated differential metabolites of *P*. *tenuiflora* seedlings under salinity after inoculation with *R*. *intraradices*. The annotated pathways of these differential metabolites were inconsistent in response to AMF inoculation under well-watered and drought stress, indicating different response mechanisms of mycorrhizal fungi promoting plants under different soil moisture conditions. Among the metabolic pathways, amino acids responded more effectively because they are the basic units of protein synthesis, and changes in them affect protein synthesis and thus interfere with normal physiological metabolism (Yang et al., 2020). AMF induced the increase in 17 amino acids (2-aminoadipic acid (L-homoglutamic acid), N-acetylmethionine, 3-(6-hydroxy-3,4-dioxo-1,5-cyclohexadien-1-yl)-L-alanine, L-glutamic acid, guanidineacetic acid (−)-3-(3,4-dihydroxyphenyl)-2-methylalanine, S-(5ʹ-adenosy)-L-homocysteine, L-pipecolic acid, L-glutamine, L-methionine methyl ester, L-alanine, N-acetyl-L-tyrosine, N-acetylaspartate, phenylalanine-phenylalanine, S-(5ʹ-adenosyl)-L-methionine, 3-hydroxy-3-methylpentane-1,5-dioic acid, and glutamic acid) and derivatives and the reduction of 3 amino acids and derivatives (3-(2-naphthyl)-D-alanine, aspartic acid-phenylalanine, and L-cysteine) under drought stress conditions. Bernardo et al. (2019) also identified 10 differential amino acids in drought-stressed wheat after AMF inoculation, 7 of which (4-amino-2-methyl-5-phosphomethylpyrimidine, 3-phospho-hydroxypyruvate, imidazole acetol-phosphate, penicillamine, L-methionine/S-ethyl-L-cysteine, O-phospho-L-tyrosine, and 3ʹ-deamino-3ʹ-oxonicotianamine) were up-regulated, and three amino acids (2-amino-3-oxobutanoate, N-acetyl-DL-methionine, and cyclogutamate) were down-regulated. In *P*. *tenuiflora* seedlings subjected to saline stress, AMF inoculation positively promoted the accumulation of 10 amino acids and amines (Yang et al., 2020). Salvioli et al. (2012) revealed the increase in the amino acid abundance in tomato fruits after inoculation with *G*. *mosseae*. In white clover, *R*. *intraradices* accelerated leaf glutamate, aspartate, arginine, and ornithine concentrations, which was associated with changes in N-assimilated enzyme activities (Xie et al., 2021). Mycorrhizal symbiosis accelerated the uptake of various amino acids in *Sorghum bicolor* such as phenylalanine, asparagine, arginine, tryptophan, etc., and the uptake of such neutral or positively charged amino acids was higher than that of negatively charged amino acids (Whiteside et al., 2012). In addition, the increase in amino acids under mycorrhization may originate from the synthesis of mycorrhizal fungi (Govindarajulu et al., 2005). More amino acids were accumulated in mycorrhizal plants after stress, implying more protein synthesis under adversity. This facilitates mycorrhizalized plants to further improve osmoregulation, maintain cytoplasmic membrane permeability and nutrients, and thus enhance the response to stress signals (Teixeira et al., 2020).

Soil drought strongly affected the terpenoid content of plants, usually showing an increasing trend in total terpenes (Turtola et al., 2003; Jiang, 2012). In our study, drought stress induced a significant accumulation of phytol in roots of non-inoculated plants as well as sweroside in inoculated plants, while inhibited the decrease in capsanthin, caryophyllene, and terpineol on non-inoculated plants. Similarly, Podda et al. (2019) also revealed the increase in phytol in *Brassica oleracea* plants in response to drought stress. It seems that phytol, a product of chlorophyll degradation, served as an important indicator of the state of chlorophyll. Other studies in *C*. *aurantium*, *Eucalyptus camaldulensis*, and *Thymus transcaucasicus* showed diverse changes in metabolites under environmental stresses (drought, salinity, and temperature stresses) conditions, along with the reduction of metabolites more commonly (Eirini et al., 2017; Manukyan, 2019; Amrutha et al., 2021). Thus, these metabolite changes are an adaptive response mechanism in trifoliate orange under drought stress.

The results of the present study showed that AMF inoculation significantly inhibited the relative concentrations of various differential metabolites in almost all cases, except for β-caryophyllene which was increased. Bernardo et al. (2019) also reported the increase of two terpenoids (a limonene-1,2-doil and (20S)-ginsenoside Rh2) and the reduction of another terpenoids (7-deoxyloganate and hemigossypol-6-methyl ether) in wheat plants under drought stress after the colonization by *F*. *mosseae*. We speculated that mycorrhizal plants possessed more biomass including roots than non-mycorrhizal plants, so there was a dilution effect, which led to the decrease in differential metabolites. Additionally, mycorrhizal fungi may have promoted effects on the amount of volatilization and release of terpenoids under drought conditions. Many terpenes are volatile organic compounds (VOCs), and a small number of studies also displayed that mycorrhizal fungal colonization increased the emission of the green leaf volatile (Z)-3-hexenyl acetate from *Plantago lanceolata* (Fontana et al., 2009) and the emission of limonene and artemisia ketone from *Artemisia annua* (Rapparini et al., 2007). The emission of these terpenoids is almost comparable to the mechanical damage and herbivore damage to plants. These suggest that mycorrhiza-induced reduction of terpenoids may lead to an increased emission of VOCs. VOCs are an indirect way for plants to attract natural enemies to defend against insect herbivores as well as for VOCs to play a signal to initiate plant defense response (Fontana et al., 2009). More work needs to be carried out around the changes in the emission of VOCs by mycorrhizal versus non-mycorrhizal plants under adversity.

Our results also displayed that AMF inoculation significantly reduced the relative concentration of phytol in roots of trifoliate orange under drought stress, but not under well-watered. In fact, in the process of abiotic stress, a certain amount of chlorophyll is disintegrated, leading to a substantial accumulation of free phytol (Podda et al., 2019). Zhang et al. (2020) also found significantly higher chlorophyll levels in drought-stressed trifoliate orange after inoculation with *F*. *mosseae*. This implies that mycorrhizal plants have a higher chlorophyll content and therefore reduced phytol levels under drought conditions. It concludes that mycorrhizal plants under drought stress are characterized with a low level of chlorophyll degradation and thus maintain a relatively high accumulation of photosynthates, which is positive for the drought tolerance of plants.

## Data Availability Statement

The original contributions presented in the study are included in the article/Supplementary Material, further inquires can be directed to the corresponding author.

## Author Contributions

FZ and Q-SW conceived and designed the experiment. FZ performed the experiments. S-ML, Y-NZ, Q-SW, and KK analyzed the data. S-ML, FZ, and KK prepared the Figures. S-ML wrote the paper. Q-SW and KK revised the paper. All authors contributed to the article and approved the submitted version.

## Funding

This study was supported by the National Key Research and Development Program of China (2018YFD1000303) and the Plan in Scientific and Technological Innovation Team of Outstanding Young Scientists, Hubei Provincial Department of Education, China (T201604). The authors are also grateful to Excelence project PrF UHK 2011/2021-2022 for the financial support.

## Conflict of interest

The authors declare that the research was conducted in the absence of any commercial or financial relationships that could be construed as a potential conflict of interest.

## Publisher’s Note

All claims expressed in this article are solely those of the authors and do not necessarily represent those of their affiliated organizations, or those of the publisher, the editors and the reviewers. Any product that may be evaluated in this article, or claim that may be made by its manufacturer, is not guaranteed or endorsed by the publisher.

## Footnotes

^1^ http://www.shimadzu.com.cn/

^2^ https://www.thermofisher.com/us/en/home/brands/applied-biosystems.html

^3^ http://www.massbank.jp/

^4^ http://kanaya.naist.jp/KNApSAcK/

^5^ ï»¿http://www.hmdb.ca/ï»¿

^6^ https://apbioinf002.wur.nl/

^7^ http://metlin.scripps.edu/index.php

^8^ http://www.kegg.jp/kegg/pathway.html
