# Supplementary figures and images for: Metabolomics Analysis Reveals Drought Responses of Trifoliate Orange by Arbuscular Mycorrhizal Fungi With a Focus on Terpenoid Profile
Source: Front Plant Sci. 2021 Oct 6;12:740524. doi: 10.3389/fpls.2021.740524 (PMC8528288; doi:10.3389/fpls.2021.740524)

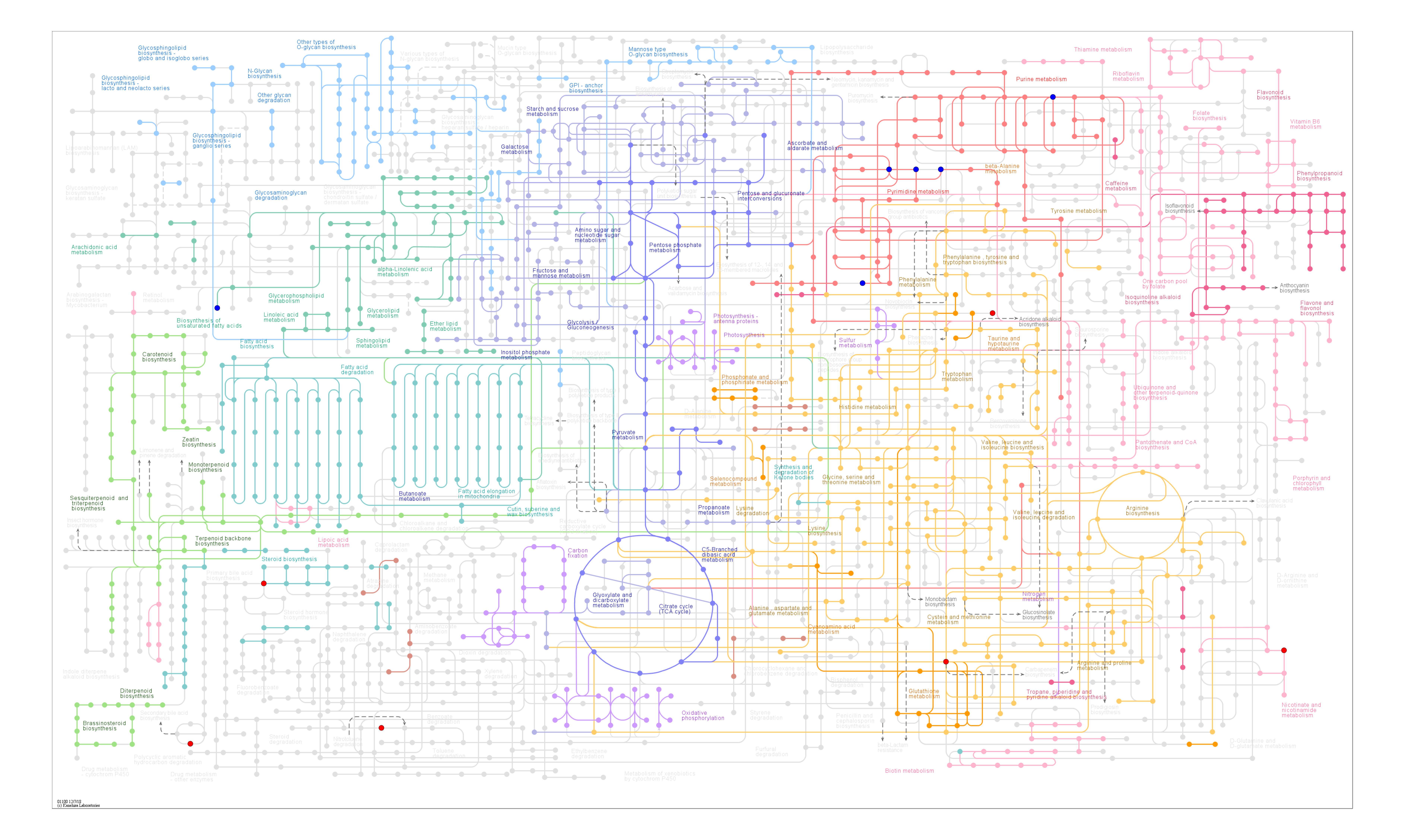

Supplement: Supplementary file 1 [file Image_1.PNG]

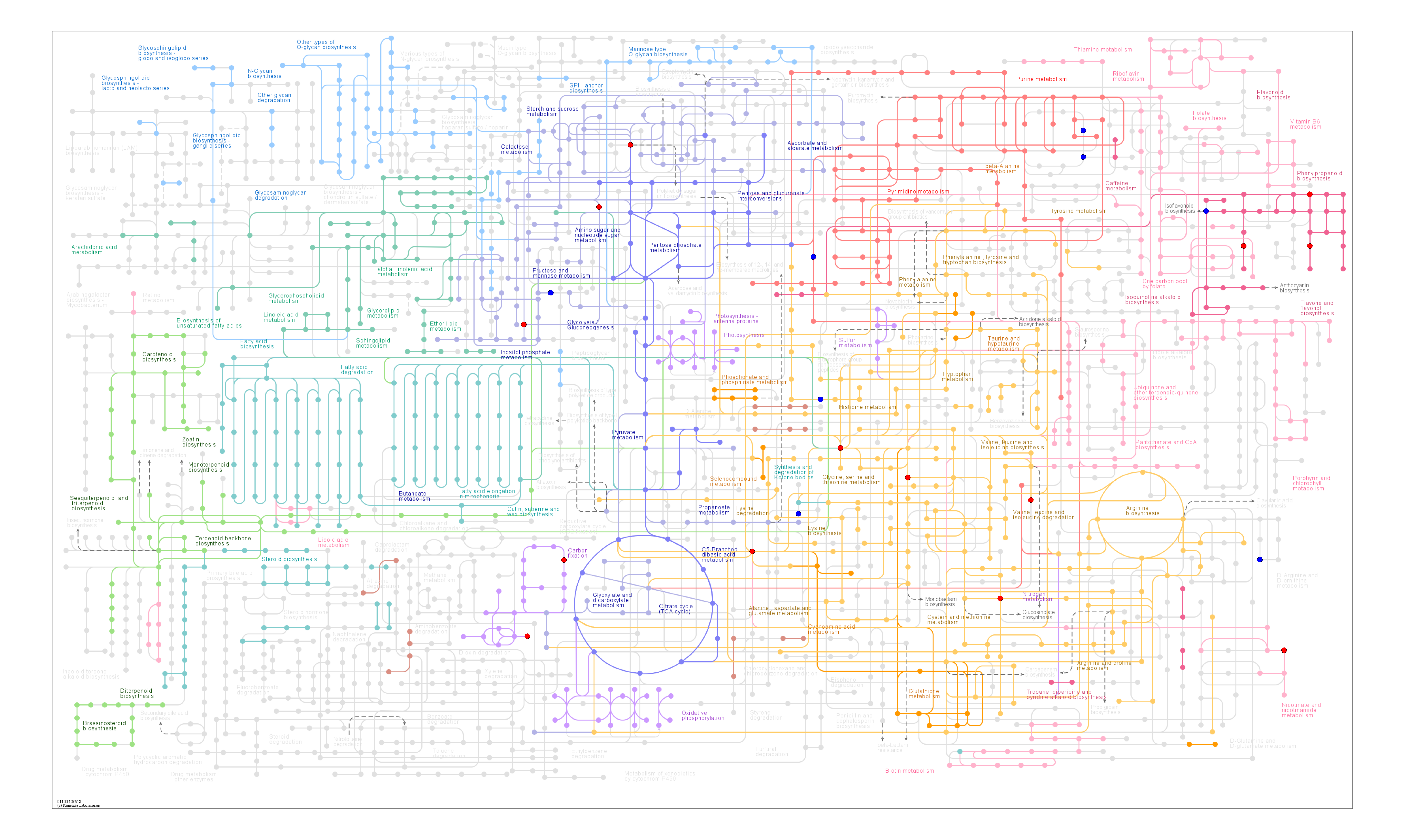

Supplement: Supplementary file 2 [file Image_2.PNG]

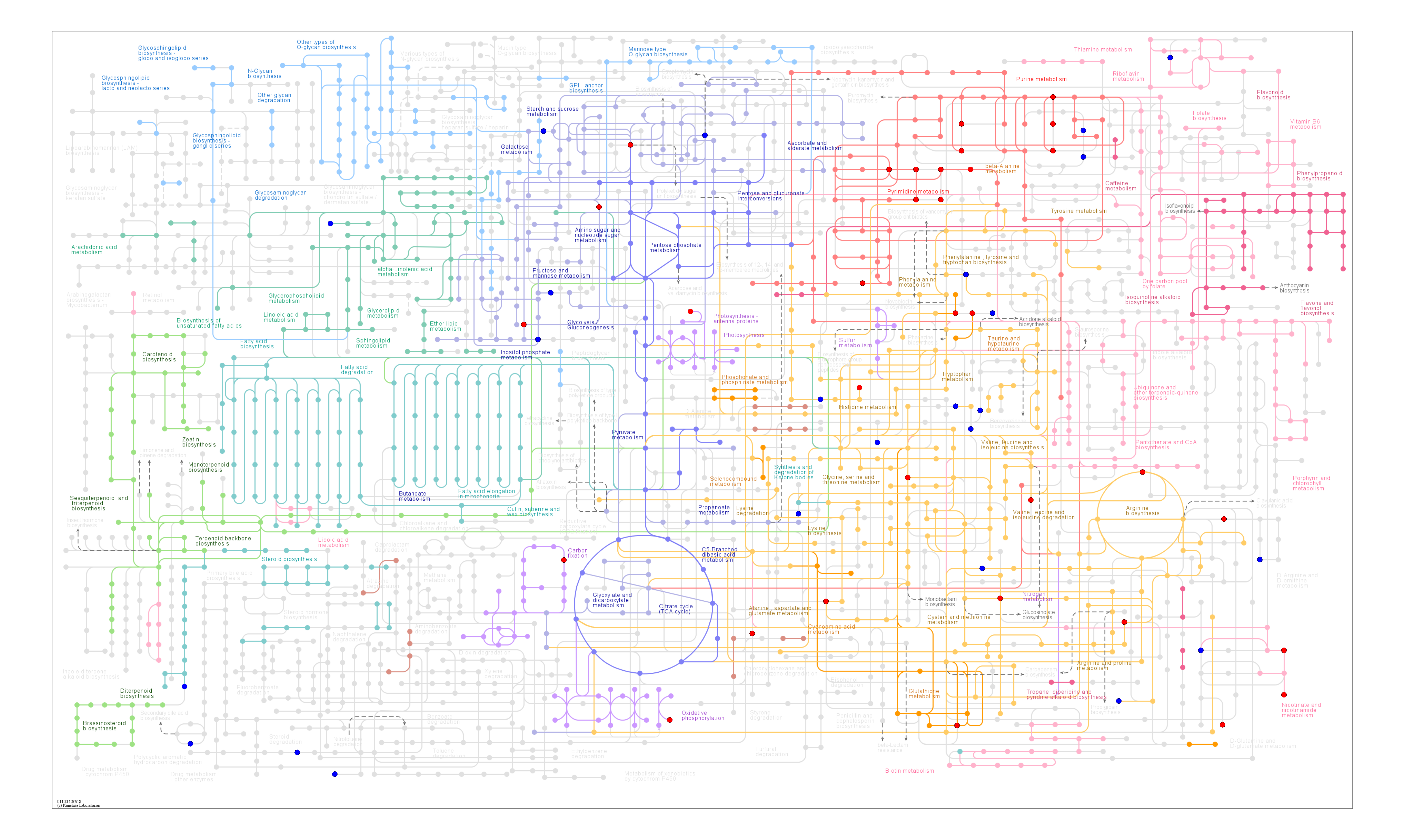

Supplement: Supplementary file 3 [file Image_3.PNG]

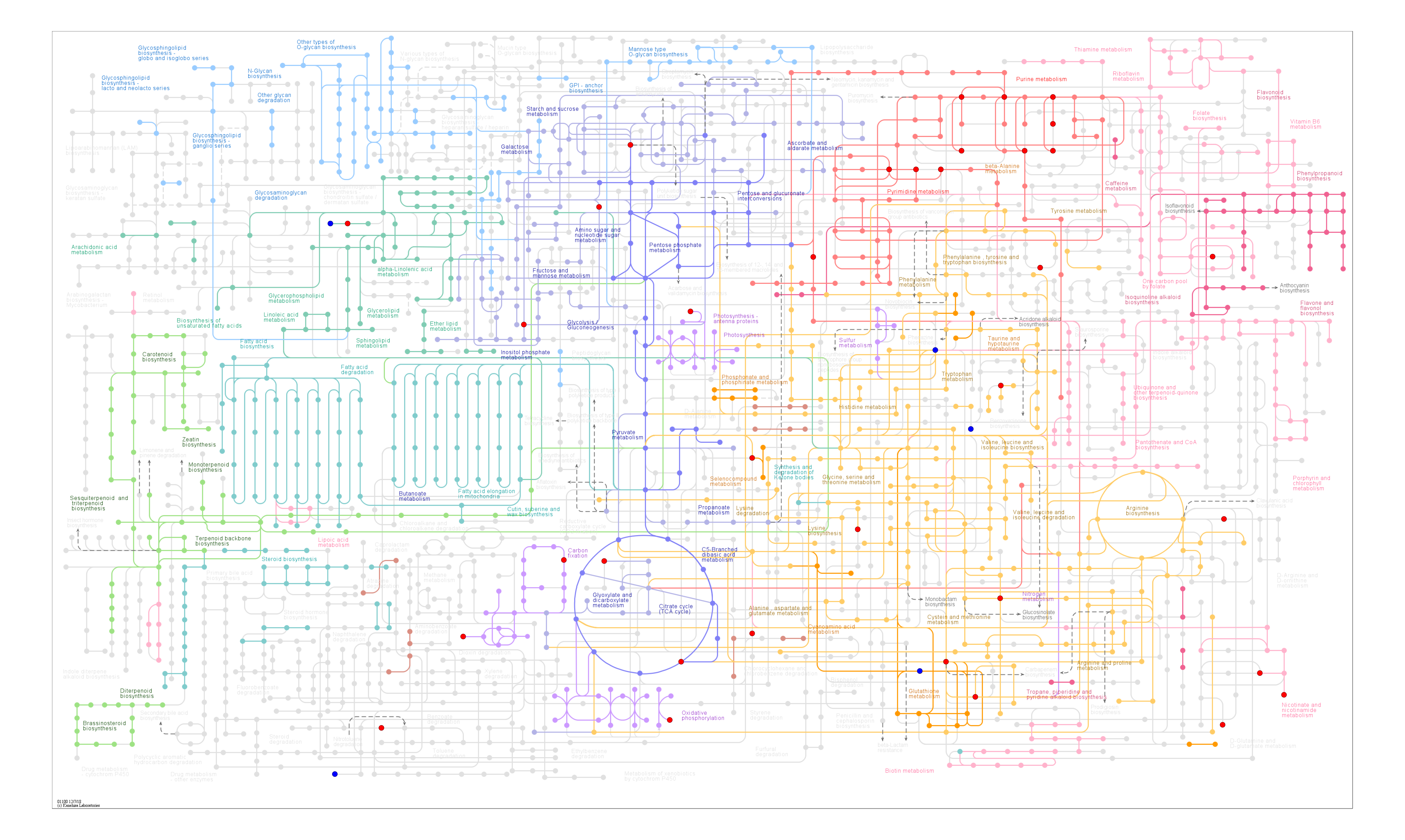

Supplement: Supplementary file 4 [file Image_4.PNG]
